# Extraction of Biomolecules Using Phosphonium-Based Ionic Liquids + K_3_PO_4_ Aqueous Biphasic Systems

**DOI:** 10.3390/ijms11041777

**Published:** 2010-04-15

**Authors:** Cláudia L. S. Louros, Ana Filipa M. Cláudio, Catarina M. S. S. Neves, Mara G. Freire, Isabel M. Marrucho, Jérôme Pauly, João A. P. Coutinho

**Affiliations:** 1 Departamento de Química, CICECO, Universidade de Aveiro, 3810-193 Aveiro, Portugal; 2 Instituto de Tecnologia Química e Biológica, UNL, Av. República, Ap. 127, 2780-901 Oeiras, Portugal; 3 Laboratoire Haute Pression Centre Universitaire de Recherche Scientifique, Université de Pau, Avenue de l’Université, 64000 Pau, France

**Keywords:** ionic liquids, aqueous two-phase systems, phase diagrams, partition coefficients

## Abstract

Aqueous biphasic systems (ABS) provide an alternative and efficient approach for the extraction, recovery and purification of biomolecules through their partitioning between two liquid aqueous phases. In this work, the ability of hydrophilic phosphonium-based ionic liquids (ILs) to form ABS with aqueous K_3_PO_4_ solutions was evaluated for the first time. Ternary phase diagrams, and respective tie-lines and tie-lines length, formed by distinct phosphonium-based ILs, water, and K_3_PO_4_ at 298 K, were measured and are reported. The studied phosphonium-based ILs have shown to be more effective in promoting ABS compared to the imidazolium-based counterparts with similar anions. Moreover, the extractive capability of such systems was assessed for distinct biomolecules (including amino acids, food colourants and alkaloids). Densities and viscosities of both aqueous phases, at the mass fraction compositions used for the biomolecules extraction, were also determined. The evaluated IL-based ABS have been shown to be prospective extraction media, particularly for hydrophobic biomolecules, with several advantages over conventional polymer-inorganic salt ABS.

## 1. Introduction

Recovery procedures usually applied to bioproducts represent the major cost associated to their extraction from natural sources. Research on more environmentally friendly and efficient techniques capable of extracting biomolecules in a single-step procedure is still ongoing. The extraction and purification of biomolecules using aqueous biphasic systems (ABS) was originally proposed by Albertsson in 1958 [1] and, in the last decades, it has been extended to the separation of cells, membranes, viruses, proteins, nucleic acids, enzymes and other added-value biomolecules [2]. Since both phases are mainly composed of water ABS are highly biocompatible processes.

Aqueous biphasic systems are usually formed by polymer-polymer, polymer-salt and salt-salt combinations in an aqueous solution. Nevertheless, in the past few years, Gutowski *et al*. [3] demonstrated that the addition of inorganic salts to aqueous solutions of ionic liquids (ILs) can cause liquid-liquid demixing and induce ABS. In addition, Abraham *et al*. [4] showed that both aqueous solutions of ILs and ABS might be regarded as novel liquid partitioning systems. As a result, ILs can be virtually used as replacements of the polymeric-rich phase in typical ABS. ILs, salts that are liquid below the temperature of 373 K, were originally suggested as alternative solvents for the replacement of the noxious volatile organic compounds currently used in industrial processes [5,6]. ILs present extremely low volatility under atmospheric conditions, and usually exhibit good thermal and chemical stability and low flammability, *i.e.,* properties that have contributed to their widespread recognition as ambient-friendly media [7–10]. The valuable properties of ILs have endorsed their use in areas ranging from organic synthesis, catalysis, extraction and separation processes, among others [11]. Moreover, the wide range of potential combinations between cations and anions allows a high degree of tunability of the ILs properties, and thus, ILs can be considered tailor-made compounds for specific tasks.

The inherent tunability of ILs allows the opportunity for the optimization of extraction processes, and although several works have been dealing with phase diagrams of IL-based ABS [12–20], their extractive potential for fundamental biomolecules has seldom been studied. Only l-tryptophan, bovine serum albumine, testosterone, epitestosterone, penicillin G, and some alkaloids have been investigated as partitioning solutes [21–29]. As a matter of fact, the potential advantages of realistic IL-based ABS have motivated previous studies by our group towards achieving a deeper understanding of the underlying molecular phenomena controlling the general picture of IL-based ABS, particularly with regard to the interactions between water and ILs [30–34], and between ILs and salts in aqueous solutions [35–39]. In spite of our contributions on the evaluation of a large rank of imidazolium-based ILs [25–29], as well as results from other authors [12,20–24], there are still many gaps in the characterization of ILs allowing the best selection of an IL for ABS formation and extraction capability. Research regarding the use of IL-based ABS has been, so far, mostly centred on the influence of several inorganic salts, or in the use of carbohydrates or amino acids, using exclusively imidazolium-based ILs [12–29]. To the best of our knowledge there are no reports concerning distinct classes of cations others than imidazolium-based ILs. This statement is of high relevance regarding the immense versatility inherent to the cation-anion permutations in ILs. Therefore, in this work, the influence of phosphonium-based ILs in promoting ABS maintaining the same inorganic salt (K_3_PO_4_) was evaluated. In this context, it must be stressed that alkylphosphonium-based salts are, in general, less dense than water - a fact that can be highly beneficial in product work-up steps for decanting aqueous streams - while imidazolium-based salts, on the other hand, are usually denser than water [40,41]. Moreover, phosphonium-based ILs are thermally more stable and have no acidic protons which make them more stable towards nucleophilic and basic conditions when compared to imidazolium- and pyridinium-based ILs [42]. Accordingly, some of these inherent characteristics of phosphonium-based ILs can be valuable for specific applications.

Different phase diagrams (binodal curves, tie-lines and tie-lines length) were determined for different hydrophilic ILs + K_3_PO_4_ + water systems, at 298 K and atmospheric pressure. The selected IL combinations allowed the study of the impact of the cation nature and the anion identity in the ABS promotion capability. In addition, the ABS investigated were characterized according to their extractive potential for distinct biomolecules ranging from food colourants, to amino acids to alkaloids, where *β*-carotene, rhodamine 6G, L-tryptophan and caffeine were selected as representative solute molecules. Besides the partition coefficient values, data on both phases’ physical properties that are necessary for the design of extraction processes are also reported. The densities and viscosities of the aqueous phases at the compositions used for the biomolecules extraction were determined in the temperature range from (298.15 to 318.15) K and compared with values presented by common polymer-inorganic salt ABS.

## 2. Experimental Section

### 2.1. Materials

**Figure 1.**
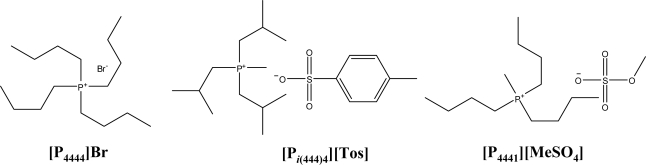
Chemical structures of the studied phosphonium-based ILs.

The ABS studied in this work were prepared using aqueous solutions of K_3_PO_4_ (≥98 wt% pure, Sigma) and aqueous solutions of individual ILs. The ILs studied were phosphonium-based ILs, namely triisobutyl(methyl)phosphonium tosylate ([P*_i_*_(444)1_][Tos]) > 95 wt % pure, tributyl(methyl)-phosphonium methylsulphate ([P_4441_][MeSO_4_]) > 99 wt % pure and tetrabutylphosphonium bromide ([P_4444_]Br) > 96 wt % pure. The studied phosphonium-based ILs were kindly provided by Cytec Industries, Inc. The selected ILs cover different cations and anions and merely represent a proof of principle of the versatility of ILs that can be used for forming ABS. All the ILs’ molecular structures and respective designations are shown in Figure 1.

For the validation of the experimental procedure adopted 1-butyl-3-methylimidazolium chloride ([C_4_mim]Cl) (>99 wt % pure, Iolitec) was used. The partitioning solutes l-tryptophan, >99.0 wt % pure, and *β*-carotene, ≥97.0 wt % pure, were obtained from Fluka. Rhodamine 6G, >95.0 wt % pure, was acquired from Merck and caffeine, ≥99.5 wt % pure, was obtained from José M. Vaz Pereira, SA. The water used was ultra-pure water, double distilled, passed by a reverse osmosis system and further treated with a Milli-Q plus 185 water purification apparatus.

### 2.2. Preparation of Phase Diagrams

The phase diagrams were determined at 298 K (±1 K) by the cloud point titration method [25,26]. Aqueous solutions of K_3_PO_4_ at 40 wt % and aqueous solutions of the different hydrophilic ILs at 80 wt % were prepared and used for the phase diagrams determination. Repetitive drop-wise addition of the aqueous inorganic salt solution to each IL aqueous solution was carried out until the detection of a cloudy and further biphasic solution, followed by the drop-wise addition of ultra-pure water until the detection of a monophasic region (clear and limpid solution). Drop-wise additions were carried out under constant stirring. The ternary systems compositions were determined by the weight quantification of all components added within an uncertainty of ± 10^−5^ g. Further details on the experimental procedure can be found elsewhere [25,26]. The experimental procedure was validated by comparison of the phase diagram obtained for the [C_4_mim]Cl and K_3_PO_4_ aqueous system against literature data [25,26], which showed them to be in close agreement.

### 2.3. Determination of Tie-Lines

Tie-lines (TLs) were determined by a gravimetric method originally proposed by Merchuck *et al*. [43]. For the determination of TLs, a mixture from the biphasic region was selected, vigorously agitated and allowed to reach the equilibrium, by the separation of both phases, for 12 h and at 298 K. After a careful separation step, both top and bottom phases were weighed. Finally, each individual TL was determined by application of the lever rule to the relationship between the top mass phase composition and the overall system composition [43]. The experimental binodal curves were fitted using Equation 1 [43]:

(1) $$Y=A\;\text{exp}\left[({BX}^{0.5})-({CX}^{3})\right]$$

where *Y* and *X,* are respectively, the IL and salt weight percentages, and *A*, *B* and *C* are constants obtained by the regression.

For the TL determination the following system of four equations (Equations 2 to 5) and four unknown values (*Y*_T_, *Y*_B_, *X*_T_ and *X*_B_) was solved [43]:

(2) $$Y_{\text{T}}=A\;\text{exp}\left[({BX}_{\text{T}}^{0.5})-({CX}_{\text{T}}^{3})\right]$$

(3) $$Y_{\text{B}}=A\;\text{exp}\left[({BX}_{\text{B}}^{0.5})-({CX}_{\text{B}}^{3})\right]$$

(4) $$Y_{\text{T}}=\left(Y_{\text{M}}/\alpha \right)-\left(\left(1-\alpha \right)/\alpha \right)Y_{\text{B}}$$

(5) $$X_{\text{T}}=\left(X_{\text{M}}/\alpha \right)-\left(\left(1-\alpha \right)/\alpha \right)X_{\text{B}}$$

where subscripts *M*, *T*, and *B* denote respectively the mixture, the top phase and the bottom phase, *X* is the weight fraction of inorganic salt, *Y* the weight fraction of IL and *α* is the ratio between the mass of the top phase and the total mass of the mixture. The system solution results in the concentration of the IL and inorganic salt in the top and bottom phases, and thus, TLs can be simply represented.

For the calculation of the tie-lines length (TLL) Equation 6 was used as follows:

(6) $$\text{TLL}=\sqrt{{\left(X_{\text{T}}-X_{\text{B}}\right)}^{2}-{\left(Y_{\text{T}}-Y_{\text{B}}\right)}^{2}}$$

where subscripts *T* and *B* denote respectively the top and bottom phases, and *X* and *Y* are the weight fraction of inorganic salt and IL, respectively. It should be pointed out that the top phase is the IL-rich phase, while the bottom phase is the K_3_PO_4_-rich phase.

### 2.4. Density and Viscosity Measurements

Measurements of viscosity and density were performed in the temperature range from 298.15 K to 318.15 K, and at atmospheric pressure, using an automated SVM 3000 Anton Paar rotational Stabinger viscometer-densimeter. Further details on the equipment can be found elsewhere [41]. The dynamic viscosity has a relative uncertainty within 0.35%, while the absolute uncertainty of the density is ±5 × 10^−4^ g·cm^−3^. Density and viscosity measurements were carried out at selected biphasic regions. Individual mixtures at the biphasic region were prepared by weight, vigorously agitated and allowed to reach the equilibrium by the separation of both phases for at least 12 h and at 298 K. The proper phase separation was noticeably indicated by the absence of turbidity in each phase. After the separation step, viscosity and density measurements were performed for both aqueous rich phases.

### 2.5. Partitioning of Biomolecules

Partition coefficients (*K*_i_) between the IL-rich and inorganic salt-rich phase were determined at 298 K for the following biomolecules: l-tryptophan (*K*_Trp_), *β*-carotene (*K*_βcarot_), rhodamine 6G (*K*_Rhod_) and caffeine (*K*_Caf_). The partition coefficients are defined as the ratio of the concentration of each biomolecule in the IL- and in the K_3_PO_4_-rich phase, and as described by Equation 7 (taken as example the biomolecule l-tryptophan):

(7) $$K_{\text{Trp}}=\frac{{\left[\text{Trp}\right]}_{\text{IL}}}{{\left[\text{Trp}\right]}_{\text{K3PO4}}}$$

where [Trp]_IL_ and [Trp]_K3PO4_ are, respectively, the concentrations of l-tryptophan in the IL and in the K_3_PO_4_ aqueous-rich phases.

**Figure 2.**
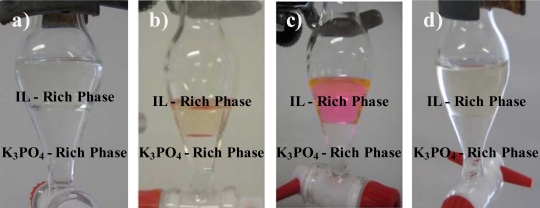
Experimental procedure applied for the biomolecules partitioning: l-tryptophan **(a),** *β*-carotene **(b)**, rhodamine 6G **(c)** and caffeine **(d)**.

A mixture in the biphasic region was selected, prepared by weight, and used to evaluate the biomolecules partitioning. For this purpose, aqueous solutions with a concentration of approximately 0.78 g·dm^−3^ (3.8 × 10^−3^ mol·dm^−3^) for l-tryptophan, 0.15 g·dm^−3^ (2.8 × 10^−4^ mol·dm^−3^) for *β*-carotene, 0.015 g·dm^−3^ (3.1 × 10^−5^ mol·dm^−3^) for rhodamine 6G and 5.0 g·dm^−3^ (2.5 × 10^−2^ mol·dm^−3^) for caffeine, were used. These aqueous solutions were used at a mass fraction composition that corresponds to the water mass fraction composition at the biphasic mixture composition selected. The solute’s initial concentration was carefully choosen after carrying out some preliminary tests aimed at achieving absorbance values in an adequate range. All biomolecules aqueous solutions can be considered at infinite dilution and completely solvated in aqueous media, thus avoiding specific interactions between themselves. The biphasic solution was left to equilibrate at (298 ± 1) K for 12 h (a time period established in previous optimization experiments) to achieve equilibrium conditions for the biomolecules partitioning between the two phases. Due care was taken with *β*-carotene, which undergoes isomerisation on exposure to light, maintaining the samples covered by aluminium foil during the time necessary for equilibration. The experimental procedure for each biomolecule extraction, as well as qualitative evidence for the coloured compounds partitioning to the IL-rich phase, is depicted in Figure 2.

The solute quantification, in both phases, was carried out by UV-Vis spectroscopy using a Shimadzu UV-1700, Pharma-Spec Spectrometer, at wavelengths of 279 nm, 512 nm, 527 nm and 274 nm for l-tryptophan, *β*-carotene, rhodamine 6G and caffeine, respectively. Calibration curves were previously established for each individual compound. All the wavelengths used for the biomolecules quantification correspond to the maximum absorption peaks of each solute. Interferences of both the inorganic salt and the IL with the analytical method were taken into account and found to not be significant at the magnitude of the dilutions performed. Three samples of each aqueous phase were precisely quantified and the respective standard deviations determined. Moreover, both phases were additionally weighted and the corresponding TLs were obtained as previously described.

## 3. Results and Discussion

### 3.1. Phase Diagrams and Tie-Lines

**Figure 3.**
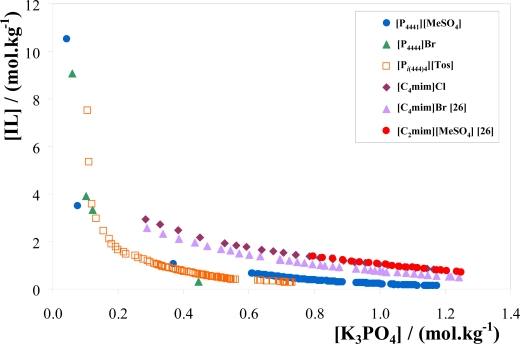
Phase diagrams for the phosphonium-based ILs and [C_4_mim]Cl ternary systems composed by IL + K_3_PO_4_ + H_2_O at 298 K.

The experimental phase diagrams for each IL + K_3_PO_4_ + H_2_O systems at 298 K and atmospheric pressure are presented in Figure 3. All data are presented in molality units for a more detailed understanding of the ILs aptitude for ABS formation (the experimental weight fraction data is provided in the Supplementary). Results obtained for the [C_4_mim]Cl IL are also presented in Figure 3 for comparison purposes. In addition, data taken from literature [26] for the ILs [C_4_mim]Br and [C_2_mim][MeSO_4_] are also included for a deeper inspection of the cation influence in promoting ABS.

Although there are several reports in the literature describing ABS comprising imidazolium-based ILs [12–29], this work is the first evidence that phosphonium-based ILs also undergo phase separation in inorganic salt aqueous systems. In addition, the studied phosphonium-based ILs display a greater ability for ABS formation than imidazolium-based ILs when evaluating both classes with similar anions. The higher the affinity for water and/or hydrophilic nature of the IL, the less effective is the IL in promoting phase separation. Therefore, the studied phosphonium-based ILs present lower affinity for water, and are thus more hydrophobic than imidazolium-based ILs. The quaternary phosphonium cations present four alkyl chains and no aromatic character, which is in turn responsible for their lower affinity for aqueous phases. Their water miscibility essentially derives from the polar water hydrogen bonding to the anions.

In Figure 3 there are small, but still visible, quantitative differences in the binodal curves for the phosphonium-based ILs. Considering, for instance, the fixed distance between the binodal curves and the origin at 0.4 mol·kg^−1^ of K_3_PO_4_, the ability of the ILs for phase separation can be described by the following order: [P_4444_]Br > [P*_i_*_(444)4_][Tos] > [P_4441_][MeSO_4_]. Thus, at such an IL concentration, [P_4444_]Br has the higher ability to induce phase separation in IL + K_3_PO_4_ + water ternary systems. Indeed this IL is the one with four straight butyl chains enhancing therefore the ABS formation ability. On the other hand, [P_4441_][MeSO_4_] presents a shorter alkyl chain coupled to a salting-out inducing anion that consequently decreases the miscibility region of the ternary phase diagram. In addition, the aromatic ring in [P*_i_*_(444)4_][Tos] is certainly the main responsible for its enhanced water affinity when compared to [P_4444_]Br. The results presented in Figure 3 show that, in general, [P_4441_][MeSO_4_] is the strongest salting-out inducing phosphonium-based IL studied while [P_4444_]Br is the strongest salting-in inducing IL.

The experimental binodal data was fitted using the approach described by Merchuck *et al*. [43] (Equation 1). The coefficients of the polynomial equation *A*, *B*, and *C* were determined by regression analysis. These values along with the correlation coefficients (*R*^2^) are given in Table 1.

**Figure 4.**
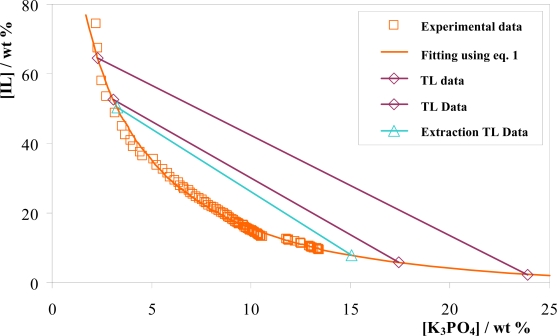
Phase diagram for the ternary system [P*_i_*_(444)4_][Tos] + K_3_PO_4_ + H_2_O at 298 K.

An example of the fitting of the experimental data using Equation 1 is provided in Figure 4. Results for the remaining ILs are provided in the Supplementary. From Figure 4 it is observed that the empirical equation correlates satisfactorily with the experimental data for the [P*_i_*_(444)4_][Tos] + water + K_3_PO_4_ system. The same analogy occurs for the remaining ILs. Therefore, Equation 1 can be useful in determining the system composition at any particular point of interest.

Tie-lines (TLs) for each ternary system were determined by the gravimetric method described previously through application of Equations 2–5. The ternary mass fraction composition of the biphasic region used to determine the TLs, TLs adjusted parameters and respective tie-lines length (TLLs) are reported in Table 2.

As an example, the results obtained for the [P*_i_*_(444)4_][Tos] + water + K_3_PO_4_ ternary system are represented in Figure 4. The TLs obtained for the ILs [P_4444_]Br and [P_4441_][MeSO_4_] are graphically represented in the Supplementary. Since the TLL represents the difference between the IL and inorganic salt concentrations in the top and bottom phases, the higher the TLL, the higher is the IL concentration in the top phase and the salt concentration at the bottom phase.

### 3.2. Density and Viscosity

**Figure 5.**
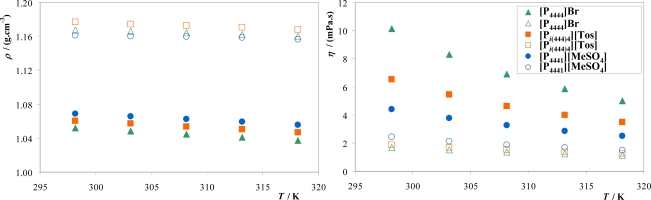
Experimental density (*ρ*) and viscosity (*η*) for the IL-rich phase (full symbols) and K_3_PO_4_-rich phase (open symbols) for systems composed by IL + K_3_PO_4_ + H_2_O at 298 K.

The physical properties of phase forming systems at various concentrations and temperatures are indispensable requirements for the design and scale up of a wide range of processes. Therefore, density and viscosity measurements were performed on both the top and bottom phases for selected mass fraction compositions at the biphasic region. The mass fraction compositions for each ternary system are presented in Table 2. The experimental data is provided in the Supplementary. Figure 5 presents the density and viscosity data as a function of temperature.

Both density and viscosity for IL- and inorganic salt-rich phases are found to decrease as the temperature increased. Typical polymer-salt systems present lower viscosities compared to polymer-polymer systems enhancing a faster segregation of the two phases after extraction procedures, which could be highly advantageous in industrial processes [43]. From the results depicted in Figure 5 no significant differences in density values between IL-based ABS and typical polymer-inorganic salt ABS are observed [44]. Similarly, the bottom phase is the inorganic salt-rich phase while the top phase is the polymer- or IL-rich phase, with density values ranging from 1.0 to 1.2 g·cm^−3^ [44]. However, larger differences are observed in viscosity values between IL-based and polymer-based ABS. IL-rich phases are far less viscous (4–11 MPa·s) than typical PEG-rich phases (39 MPa·s) at mass fraction compositions close to those measured in this work [44]. Therefore, since IL-based ABS are less viscous it constitutes a major advantage for the mass transfer process between the phases and phase handling. The lower viscosity of IL-based ABS compared with polymer-based ABS is thus very important from an industrial point of view.

The density of the inorganic salt-rich phase is higher than the corresponding equilibrated IL-rich phase. [P_4441_][MeSO_4_] is the IL studied with smaller differences in density values among both phases. The density differences among phases generally increase with the increase of the TLLs since larger differences in the mixture composition are occurring. Indeed for [P_4441_][MeSO_4_] the TLL, at the mixture composition selected, is the shorter one. On the other hand, viscosities of the IL-rich phase, and at the mass fraction compositions evaluated, decrease in the rank: [P_4444_]Br > [P*_i_*_(444)4_][Tos] > [P_4441_][MeSO_4_]. Nevertheless, the opposite order is verified for the inorganic-salt rich phases. Both differences in viscosities and densities at the K_3_PO_4_-rich phase indicate that there is some IL migration for this phase contributing for these deviations. However, differences among the inorganic salt-rich phases are significantly smaller than differences between distinct IL-rich phases. In fact, the trend on viscosities for the K_3_PO_4_-rich phases follows the trend on the IL affinity for water. From Figure 3, [P_4441_][MeSO_4_] is the phosphonium-based IL with an higher affinity for water and as a consequence it will partition in a more pronounced way to the inorganic salt-rich phase, enhancing thus the viscosities of such phase.

### 3.3. Partitioning of Biomolecules

Figure 2 provides qualitative evidence for the preferential migration of the coloured compounds to the IL-rich phase. The success of the extractive potential of ABS largely depends on the ability to manipulate phase properties aimed at obtaining appropriate partition coefficients and specific selectivities for the biomolecules of interest. There are several approaches to manipulate the partitioning of the solute in the studied systems, such as the application of different salting-out inducing salts and/or ILs, changing either the concentration of salt or IL, and the introduction of additional co-solvents, anti-solvents or amphiphilic structures. Therefore, the inorganic salt and IL compositions selected for the biomolecules partitioning, as well as the respective partition coefficients, TLs and TLLs are reported in Table 3.

From the results obtained it can be established that the addition of biomolecules into the water phase, at least at low enough concentrations, has no influence in the TLs and TLLs [26], and as graphically shown in Figure 4. In fact, these TLs and TLLs can be considered additional phase equilibrium data. These trends are in close agreement with previous results from our group [26].

During the partitioning of l-tryptophan, rhodamine 6G, *β*-carotene and caffeine there are several competing interactions between the IL, the inorganic salt, the biomolecules and water. Hydrogen-bonding, π··· π interactions, dispersive interactions, as well as electrostatic interactions between different compounds, are some examples of these interactions.

l-Tryptophan, rhodamine 6G and caffeine are considered quite hydrophilic biomolecules, while *β*-carotene is highly hydrophobic with a negligible solubility in water [45]. For rhodamine 6G the results indicate that the partitioning behaviour increased with the IL hydrophilic nature, being [P_4441_][MeSO_4_] the most efficient in its extraction, followed by [P*_i_*_(444)4_][Tos], and finally by [P_4444_]Br that presents indeed a negligible *K*_Rhod_. Except for [P_4444_]Br, rhodamine 6G preferentially partitions to the IL-rich phase (*K*_Rhod_ > 1). The trend on the ILs’ extraction capability for fairly hydrophilic solutes follows their phase behaviour and affinity for water, and as depicted in Figure 3. In addition, for the IL [P_4441_][MeSO_4_] the solutes affinity contribution in partitioning follows the order: l-tryptophan ≈ rhodamine 6G > caffeine. Nevertheless, comparing with previous results from our group using imidazolium-based ILs [25–27], the partition of l-tryptophan and caffeine are significantly lower when using the evaluated phosphonium-based ILs. For example, with the [C_4_mim]Cl + K_3_PO_4_ + water system the *K*_Caf_ is ≈ 49 [27] while *K*_Tryp_ is ≈ 37 [25] (for a mass fraction composition of 25 wt % of IL and 15 wt % of K_3_PO_4_ at 298 K). This trend clearly reflects the strong hydrophobic nature of the studied phosphonium-based ILs and their lower ability for extracting hydrophilic solutes. Indeed, while imidazolium-based ILs present higher partition coefficients of caffeine compared to l-tryptophan, the opposite is verified using the phosphonium-based ILs. On the other hand, and since *β*-carotene is highly hydrophobic, its extraction is highly efficient with [P*_i_*_(444)4_][Tos], achieving partition coefficients in the order of 61. In effect, the partition coefficient of *β*-carotene is highly significant compared to the remaining biomolecules.

In summary, it can be established that high *K*_i_ values can be obtained for hydrophobic solutes using phosphonium-based ABS, and that these systems may be a successful and a clean approach for biomolecule separation and purification in biotechnological processes. Furthermore, the large range obtained in the partition coefficients values by changing the IL indicates that the individual biomolecules extraction efficiency can be manipulated by the correct choice of the IL cation and/or anion.

## 4. Conclusions

The ability of hydrophilic ILs to form salt–salt ABS allows them to be used in aqueous separation systems. It was here shown, for the first time, that phosphonium-based ILs are also able to form ABS in the presence of the inorganic salt K_3_PO_4_. The novel phase diagrams for the ternary systems composed by IL + water + K_3_PO_4_, at 298 K and atmospheric pressure, were determined and presented. In the presence of similar anions, the studied phosphonium-based ILs have been shown to be more effective in promoting ABS compared to the typical imidazolium-based class. The capacity of phosphonium-based ABS as prospective extraction media in biotechnological processes was demonstrated by the high partition coefficients obtained with hydrophobic biomolecules, such as *β*-carotene. Indeed, the partitioning behaviour of all studied biomolecules follows the hydrophilic/lipophylic balance between the ILs and themselves. Moreover, these IL-based ABS are shown to present lower viscosities than typical polymer-based ABS that is highly relevant for the mass transfer enhancement between both aqueous phases and their proper manipulation.

## Figures and Tables

**Table 1.** Correlation parameters of Equation 1 used to describe the experimental binodal data.

**IL + K**_**3**_**PO**_**4**_**+ Water system**	***A***	***B***	***C***	***R***^***2***^
[P_4444_]Br	176.57	−0.7562	1.100 × 10^−3^	0.9987
[P*_i_*_(444)4_][Tos]	229.48	−0.8378	3.614 × 10^−5^	0.9863
[P_4441_][MeSO_4_]	116.85	−0.5131	1.217 × 10^−4^	0.9864

**Table 2.** Experimental mass fraction composition, TLs and respective TLLs.

**IL + K**_**3**_**PO**_**4**_**+ Water system**	**Weight fraction composition/wt %**		**TL equation[a]**		**TLL**

	**IL**	**K**_**3**_**PO**_**4**_	***a***	***b***	
[P_4444_]Br	40.98	5.930	71.70	−5.181	62.38
	49.95	6.516	79.54	−4.542	75.21
	40.12[b]	6.010[b]	70.96	−5.132	61.47

[P*_i_*_(444)4_][Tos]	39.71	10.90	71.07	−2.878	65.77
	30.07	9.980	62.76	−3.276	49.04
	39.93[b]	6.062[b]	62.04	−3.648	43.38

[P_4441_][MeSO_4_]	22.98	9.986	48.60	−2.566	24.88
	20.13	15.11	57.16	−2.451	51.80
	22.38[b]	10.61[b]	48.46	−2.458	28.75

[a] IL (wt %) = *a* (wt %) + *b* × K_3_PO_4_ (wt %)

[b] Mass fraction composition at which the viscosities and densities were determined

**Table 3.** Mass fraction compositions and partition coefficients of L-tryptophan, *β*-carotene, rhodamine 6 G and caffeine in IL + K_3_PO_4_ + H_2_O systems at 298 K.

**IL + K_3_PO_4_ + Water system**	**Weight fraction composition/wt %**		**TL equation[a]**		**TLL**	***K*_i_**

	**IL**	**K_3_PO_4_**	***a***	***b***		
**l-tryptophan**						

[P_4441_][MeSO_4_]	22.95	10.66	50.39	−2.574	31.86	9.0 ± 0.1

***β*-carotene**						

[P*_i_*_(444)4_][Tos]	39.62	6.516	64.22	−3.776	46.04	61 ± 5

**rhodamine 6G**						

[P_4444_]Br	39.92	5.995	72.86	−5.494	62.73	0.018 ± 0.007
[P*_i_*_(444)4_][Tos]	40.11	6.118	62.23	−3.615	44.14	3.6 ± 0.1
[P_4441_][MeSO_4_]	23.66	10.22	48.61	−2.442	29.94	8 ± 1

**caffeine**						

[P_4441_][MeSO_4_]	23.62	10.26	49.49	−2.521	30.38	4.75 ± 0.01

[a] IL (wt %) = *a* (wt %) + *b* × K_3_PO_4_ (wt %)
